# Gelatin-Based Antimicrobial Films Incorporating Pomegranate (*Punica granatum* L.) Seed Juice by-Product

**DOI:** 10.3390/molecules25010166

**Published:** 2019-12-31

**Authors:** Arantzazu Valdés, Esther Garcia-Serna, Antonio Martínez-Abad, Francisco Vilaplana, Alfonso Jimenez, María Carmen Garrigós

**Affiliations:** 1 Department of Analytical Chemistry, University of Alicante, Nutrition & Food Sciences, ES-03690 San Vicente del Raspeig, Alicante, Spain; arancha.valdes@ua.es (A.V.); esther_gsg@outlook.com (E.G.-S.); antma@ua.es (A.M.-A.); mc.garrigos@ua.es (M.C.G.); 2 Division of Glycoscience, School of Biotechnology, KTH Royal Institute of Technology, AlbaNova University Centre, 114 21 Stockholm, Sweden; franvila@kth.se

**Keywords:** active packaging, antimicrobial, fish gelatin, *Punica granatum* L. seed juice by-product, edible film

## Abstract

Pomegranate (*Punica granatum* L.) seed juice by-product (PSP) was added as reinforcing and antimicrobial agent to fish gelatin (FG) films as a promising eco-friendly active material for food packaging applications. A complete linkage analysis of polysaccharides in PSP showed xylan and cellulose as main components. This residue showed also high total phenolic content and antioxidant activity. Three formulations were processed by adding PSP to FG (0, 10, 30 wt. %) by the casting technique, showing films with 10 wt. % of PSP the best performance. The addition of PSP decreased elongation at break and increased stiffness in the FG films, particularly for 30 wt. % loading. A good compatibility between FG and PSP was observed by SEM. No significant (*p* < 0.05) differences were obtained for barrier properties to oxygen and water vapour permeability compared to the control with the incorporation of PSP, whereas water resistance considerably increased and transparency values decreased (*p* < 0.05). High thermal stability of films and inhibition against *S. aureus* were observed. The addition of PSP at 10 wt. % into FG was shown as a potential strategy to maintain the integrity of the material and protect food against lipid oxidation, reducing huge amounts of pomegranate and fish wastes.

## 1. Introduction

Fish gelatin (FG) has been recently proposed as an excellent bio-based and biodegradable matrix in active food packaging, replacing conventional non-biodegradable polymers and other mammalian-based gelatins, increasing its commercial potential for specific groups of consumers, such as vegetarian and kosher food [1]. FG protein-based films are tasteless, colourless, transparent, water-soluble, show excellent barrier properties to oxygen and carbon dioxide being able to inhibit lipid oxidation in foodstuff, and present higher flexibility properties than other bio-based films for food packaging [2]. In addition, FG has shown great potential as an excellent matrix to host bio-active compounds, such as carvacrol [3], boldine [4], tea polyphenols [5], and olive phenols [6], giving enhanced functionalities, such as antioxidant/antimicrobial, to FG-based formulations.

Since FG is mainly obtained from fish skins and bones, the benefits of its use include the reduction of fishery wastes, contributing to the well-known circular economy concept [7]. In fact, around 30% of residues and by-products are obtained during fish processing [8] which results in huge amounts of fish wastes produced every year to be discarded considering that the total production of fishery products in Europe was 6.3 million tonnes in 2016 [9], underlining the need for searching new sustainable alternatives to reduce fish production waste.

Nevertheless, FG edible films show two main disadvantages, conditioning their use in the production of materials for food packaging. FG is highly hygroscopic, showing poor water resistance and low mechanical strength [10]. Indeed, cellulose nanofibers [11], nano-SiO_2_ particles [12], montmorillonite [13], coconut husk [14], or chitosan nanoparticles [15] have been proposed as reinforcing agents in FG matrices. Another interesting approach was recently proposed by using Maillard reactions induced by heating FG films with glucose resulting in the increase in tensile strength, elongation at break and glass transition temperatures [16].

Over the last decade, pomegranate (*Punica granatum* L.) fruit has been reported to exert a promising preventive activity against several inflammatory and chronic diseases such as cancer, cardiovascular diseases and diabetes [17]. It is extensively used in several food products such as jams, infusions and juices among others [18]. The main pomegranate production area in Europe is located in the eastern part of Spain, particularly in the Valencian Community, reaching a total annual production of 50 thousand tonnes [19]. The non-edible fractions of the mesocarp (rings, arils and seeds) contain high concentrations of hydrolysable tannins (32–263 g/kg) and isolariciresinol (5.0–13.6 mg/kg) [20]. The main composition of pomegranate by-products as well as their potential to enhance specific functionalities in food applications have been recently reviewed [21]. Seeds comprise around 10% of the total fruit weight, depending on the climatic conditions for growing as well as the maturation degree on harvesting [22]. Pomegranate seeds are a rich source of total unsaturated fatty acids (punicid, linolenic and linoleic acids), proteins, minerals, vitamins, pectin, isoflflavones (mainly genistein) and other polyphenols [23].

The extraction of value-added chemicals and compounds from pomegranate peels has been recently reported by some authors [24,25,26,27]. In addition, the use of pomegranate peel, seed oil and seed extract in film formulations has been investigated [28,29,30,31]. However, at the best of our knowledge, no available studies have been reported regarding the direct use of pomegranate seeds and arils by-products into biopolymer matrices. Therefore, the aim of this study is the development of new antimicrobial FG-based bio-films reinforced with pomegranate (*Punica granatum* L.) seed juice by-products (PSP), pressed seeds and arils, with potential application in active packaging. A full characterization of PSP to increase it added-value potential was carried out. Furthermore, the effect of PSP addition on the optical, morphological, structural, thermal, mechanical, barrier and antimicrobial properties of the developed FG/PSP films containing different PSP loadings was investigated.

## 2. Results

### 2.1. PSP Characterization

The chemical composition obtained for PSP showed humidity, ash and fat contents of 4.51 ± 0.25, 1.82 ± 0.12 and 16.26 ± 0.37 g/100 g, respectively. Higher contents were obtained by Gül and Şen when studying the potential of pomegranate seed flour (PSF) as a functional ingredient in wheat bread [32], reporting values of 10.67% for moisture, 3.21% for ash, and 18.24% for total lipid contents in PSF. These differences could nevertheless be expected since PSP is the result of the mixture of pressed seed and arils and not only seeds and, therefore, compositions should be different.

The sugar composition of PSP determined after trifluoroacetic acid (TFA) hydrolysis and a two-step sulfuric hydrolysis is shown in Table 1. Results obtained from each hydrolysis procedure are in good agreement with previously reported results for pomegranate peel [33,34]. To the best of our knowledge, for the first time, a complete linkage analysis of polysaccharides was also performed to investigate the type, structure and abundance of polysaccharides in PSP (Table 2). As TFA hydrolysis is not able to digest crystalline cellulose, a good estimation of the cellulose content can be obtained by subtracting the glucose value to the total glucose, calculated by sulfuric hydrolysis [35] (Table 1). The main constituents found in PSP were xylan and cellulose, comprising more than 80 wt. % of the total polysaccharides content. The main structure of xylan present in PSP is a typical glucuronoxylan, comprising β-1→4 linked xylose units partially substituted at mainly C-2 with 4-*O*-methyl-glucuronosyl (MeGlcA) side groups (Table 2). 

The degree of glucuronosyl substitution is, however, relatively low compared to other common sources, such as sugarcane bagasse and straw [36]. A lower degree of substitution can be ascribed to the higher crystallinity and lower solubility of xylan, which should directly impact in an improvement in mechanical reinforcement capacities and barrier properties of PSP [37]. Both, cellulose and xylan content in PSP, would in this way contribute to increase its reinforcing potential in edible films or other food packaging applications. An increase in brittleness due to this high crystallinity might be somewhat balanced in PSP by the high pectin content (around 10 wt. %). This comprises rhamogalacturonans, arabinogalactans, homo-galacturonan and arabinan (Table 2). Finally, β-1→4 linked mannose units infer the presence of relatively low quantities of mannan.

PSP showed a total phenolic content (TPC) value of 73.8 ± 0.5 mg gallic acid equivalent (GAE)/g. This result is higher than those reported for pomegranate peel extracts obtained from the co-product of juice extraction with TPC values of 54.54 [38] and 19.30 mg GAE/g [24], and pomegranate seed flower with TPC of 40.60 mg GAE/g [32], respectively. However, a TPC value of 81.15 mg GAE/g was reported by Harini et al. from pomegranate peel ethanolic extracts [39]. Regarding antioxidant activity, the extract concentration required to cause 50% reduction in the initial concentration (IC_50,_ mg/mL) values obtained from 2,2-diphenyl-1-picrylhydrazyl (DPPH) and 2,2-azinobis(3-ethyl-benzothiazoline-6-sulphonate) (ABTS) methods were 3.81 ± 0.05 and 3.95 ± 0.04 mg/mL extract, respectively. These results are higher than those reported for pomegranate seed flour with an IC_50_ of 1.05 mg/mL (DPPH) [32] and pomegranate peels extracted with ethanol showing IC_50_ values of 3.132 µg/mL (DPPH) and 5.013 µg/mL (ABTS) [40]. These differences found among reported results in TPC and antioxidant activity have been related to variability in cultivars, climate and soil conditions and maturity [32]. Moreover, the concentration of phenolics varies depending on the different parts of the fruit, showing peel extracts higher levels of antioxidant activity compared to seeds and juices. In this sense, pomegranate peel extracts have been reported to be rich in flavonols, flavanones, flavones, ellagic acid, gallic acid, ellagitannins and anthocyanidins; whereas pomegranate seed extracts have been reported to be a source of non-steroid phytochemicals and fatty acids, i.e., punicic acid, linoleic acid, oleic acid, palmitic acid and stearic acids [41]. Derakhshan et al. found TPC values ranging from 276–413 mg GAE/g and 72.4–73.0 mg GAE/g for pomegranate peel and seed ethanolic extracts, respectively when studying three different Iranian cultivars [42]. Manasathien et al. reported TPC and IC_50_ (DPPH) values of 449.6 mg GAE/g and 121.6 µg/mL for pomegranate peel ethanolic extracts, respectively; and 77.93 mg GAE/g and 1324.35 µg/mL for pomegranate seed ethanolic extracts, respectively [41]. These results are in line with those obtained in this work considering that the sample used was a mixture of seeds and arils by-products from two mixed Spanish varieties such as Mollar and Wonderful [43]. The extraction process has also a significant impact on the antioxidant activity. In the present work, ethanol was used as a sustainable solvent which has been also reported to give higher extraction yields from pomegranate peels and seeds compared to methanol and ethyl acetate [44] or water [41]. Finally, antioxidant activity for PSP determined by FRAP was 0.91 ± 0.07 mM TE/g which is in accordance with values obtained in literature for pomegranate peels (0.99 mM TE/g) [38]. The obtained results suggest that PSP waste has a high level of phenolic content and antioxidant activity, being a potential antioxidant source.

### 2.2. Thickness, Transparency and Morphology of FG/PSP Films

Some increase in film thickness was observed with increasing PSP content, obtaining 102 ± 8 µm, 132 ± 6 µm and 162 ± 8 µm for FG, FG/PSP 10 and FG/PSP 30, respectively. Hanani et al. found similar results when incorporating different contents of pomegranate peel powder (PPP) to FG films and this behaviour was attributed to the non-complete solubilisation of PPP in the film-forming solution and the presence of insoluble particles in the films [28].

Transparency is one of the most relevant properties of films in terms of overall appearance and consumer acceptance. Optical density results were obtained in the ultraviolet and visible regions (λ = 280 nm and λ = 600 nm) for all tested materials. It should be noted that higher optical density values at both wavelengths represent higher opacity of the material since the transmission of light through the film is lower. In this case, gelatin films incorporated with PSP showed a significant increase (*p* < 0.05) in optical density values with increasing PSP concentration, indicating higher opacity of films due to the dry matter content of PSP, which could difficult light transmission through the films (Table 3). Consequently, gelatin films containing 30 wt. % exhibited the best light barrier properties, providing good protection against UV and visible light, which can be an advantage to protect food products susceptible to lipid oxidation [14]. Other authors have also reported this behaviour, in agreement with our results, for different edible films containing pomegranate peel, seed oil or tomato pomace by-products [29,30,45].

**Figure 1:**
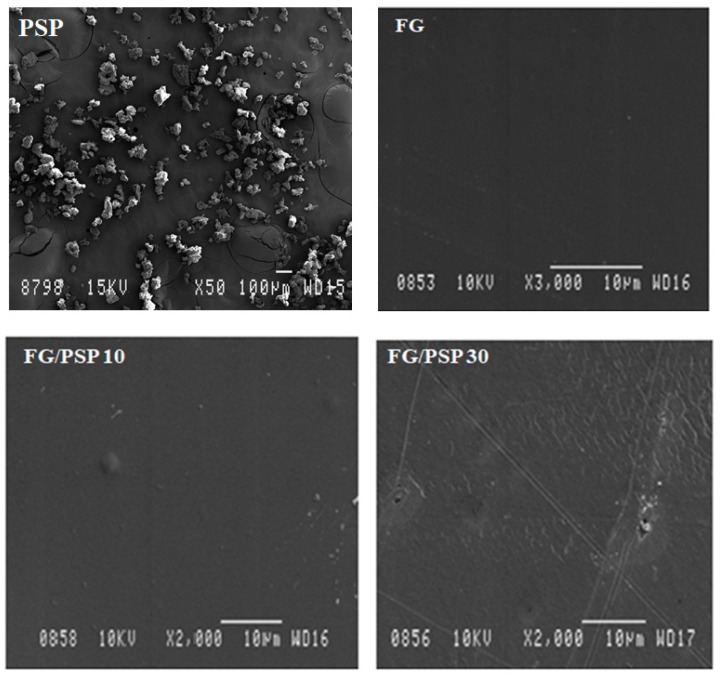
SEM micrographs of the surface of PSP and fish gelatin films containing PSP.

A homogeneous granular microstructure was observed for PSP (Figure 1) by scanning electron microscopy (SEM) with dimensions of PSP particles ranging from 20 to 80 µm in width, with an average value of 40 ± 3 µm. 

Regarding length, dimensions ranging from 40 to 80 µm with an average of 60 ± 3 µm were obtained. These results are in agreement with previous works dealing with similar materials, such as okra [46], curaua fibres [47] and almond skin [48] used as reinforcing agents in polymer composites. The surface morphology of neat FG and FG/PSP films was also studied by SEM. A smooth and homogeneous surface without pores was observed in the control film (FG) (Figure 1) showing an excellent structural integrity with no apparent heterogeneities. However, some heterogeneity was observed in the surface of FG/PSP films, being more evident in the FG film containing 30 wt. % of PSP, which was related to the PSP dispersion into the FG matrix. Similar results were reported by other authors in gelatin and starch films incorporated with pomegranate peel [28,29], and this behaviour was attributed to the presence of insoluble filler particles embedded in the films.

### 2.3. Attenuated Total Reflectance-Fourier Transform Infrared (ATR-FTIR) Spectroscopy Analysis

**Figure 2:**
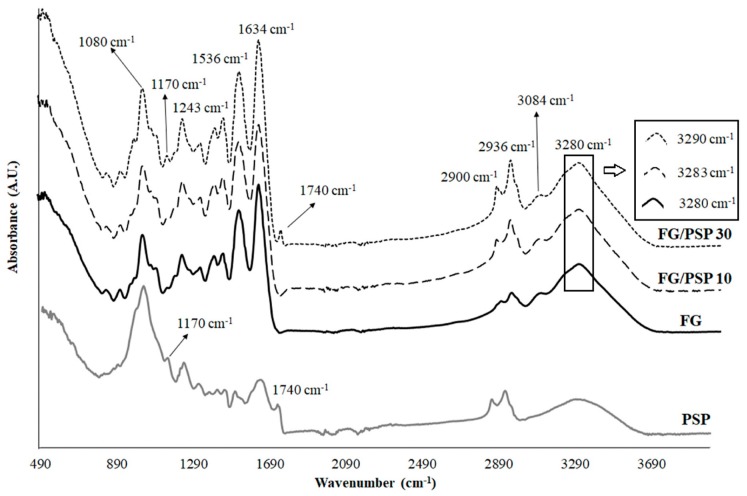
ATR-FTIR spectra of PSP and fish gelatin films containing PSP.

Figure 2 shows the ATR-FTIR spectra obtained for PSP and FG/PSP films. Some characteristic bands of PSP related to specific functional groups were observed. The band around 3280 cm^−1^ was related to the O-H stretching vibration and the hydrogen bonds in hydroxyl groups of cellulose, hemicelluloses, lignin and antioxidants [47,49]. The bands at 2936 and 2900 cm^−1^ were correlated to symmetric C-H stretching vibrations of cellulose and asymmetric C-H stretching vibrations of hemicelluloses, respectively [49]. The C=O stretching of lignin and hemicelluloses was identified by the band appearing around 1740 cm^−1^ [49], whereas the band around 1634 cm^−1^ was related to the asymmetric COO^−^ stretching of hemicelluloses. Other bands around 1536 cm^−1^ and 1243 cm^−1^ were associated to the C=C-C aromatic ring and the C-O stretching vibration of the acetyl group in lignin and hemicelluloses, respectively [49]. Finally, the two peaks at 1170 cm^−1^ and 1082 cm^−1^ were associated to the C-O-C stretching vibration of the pyranose ring in polysaccharides [47].

ATR-FTIR spectra for FG/PSP films showed similar characteristic bands at approximately 3280, 1634, 1536 and 1243 cm^−1^ which could be attributed to amide A, amide-I, amide-II and amide III groups, respectively [50,51]. Peaks observed at 3084, 2936 and 2900 cm^−1^ can be attributed to the -NH stretching and asymmetric -CH stretching vibrations and symmetrical and asymmetrical stretching of the aliphatic C-H in methylene and methyl groups, respectively.

According to the composition provided by the FG supplier, it is expected that 18 amino acids should be present, with different hydrophobic/hydrophilic character and charge, mainly the following: hydrophobic glycine (34.7 wt. %), hydrophobic alanine (10.9 wt. %), hydrophobic proline (9.8 wt. %), negatively charged glutamic acid (6.8 wt. %), hydrophilic serine (6.3 wt.%), positively charged arginine (6.2 wt. %), hydrophilic hydroxyproline (5.5 wt. %), aspartic acid (3.7 wt. %), threonine (3.3 wt. %), lysine (3.2 wt. %) and other amino acids in minor quantities. It was reported that these amino acids could allow the development of a three dimensional network structure of protein molecules by the establishment of hydrophobic and hydrogen interactions [52]. The addition of PSP to FG could modify this ordered network observing some appreciable structural changes in the ATR-FTIR spectra. In this sense, the wavenumber of amide-A peak was shifted to higher frequencies (from 3280 cm^−1^ for FG control to 3283 and 3290 cm^−1^ for FG/PSP10 and FG/PSP30 films, respectively), as observed in the zoom region in Figure 2, suggesting that PSP induced some interaction between phenolic compounds present in this residue and NH_2_ group of gelatin. A similar behaviour was observed by Hoque et al. in gelatin films incorporated with different polyphenolic extracts suggesting a cross-linking of gelatin, and the establishment of hydrophobic interactions between the hydrophobic groups of polyphenols and the hydrophobic region of FG [53]. In addition, it has been reported that functional groups in PSP, constituting cellulose, hemicelluloses and polyphenols, could act as hydrogen donors, promoting the formation of hydrogen bonds between the individual fibres [14]. On the other hand, another main difference in the ATR-FTIR spectra of FG/PSP films was observed for PSP bands at 1740 cm^−1^, related to lignin and hemicelluloses components, and bands at 1170 cm^−1^ and 1082 cm^−1^ associated to the vibration of the pyranose ring in polysaccharides. In general terms, these bands tend to increase with increasing PSP content [51].

### 2.4. Thermal Properties of FG/PSP Films

**Figure 3:**
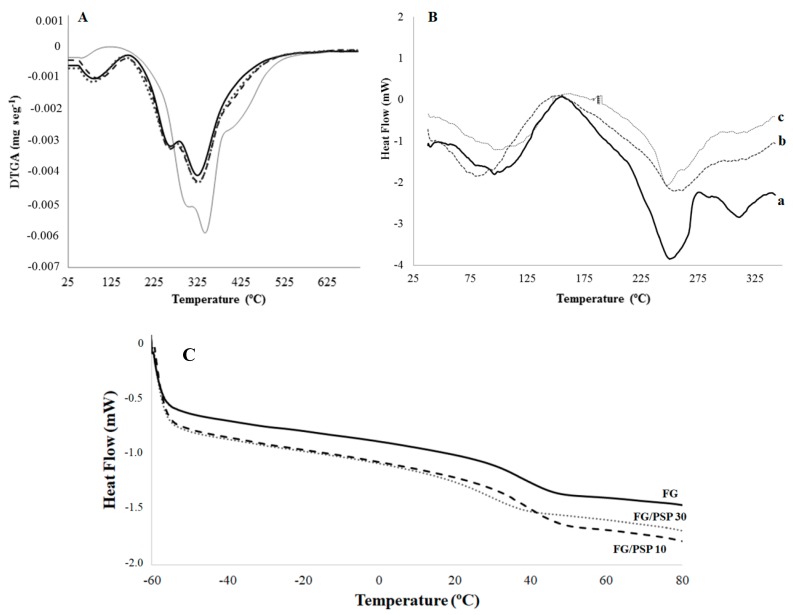
(**A**) DTGA curves of PSP (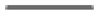), FG (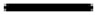), FG/PSP10 (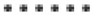) and FG/PSP30 (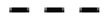) films. (**B**) DSC thermograms of FG (**a**), FG/PSP10 (**b**) and FG/PSP30 (**c**) films. (**C**) T_g_ values obtained from the second scan of DSC curves for FG, FG/PSP10 and FG/PSP30 films.

Differential thermogravimetric analysis (DTGA) curves of PSP and FG/PSP films are shown in Figure 3A. PSP showed three main degradation steps characteristic of the thermal degradation of hemicelluloses (304 ± 2 °C), cellulose (343 ± 1 °C) and lignin (396–562 °C), respectively [48]. The amplitude of the lignin band can be explained by its complex cross-linked structure with high molecular weight causing a slow thermal degradation process covering a broad temperature range [54]. On the other hand, FG films showed three characteristic main degradation stages. The first one was related to the loss of moisture, bound water and volatile compounds at temperatures lower than 100 °C. The second step (T_max_ = 259 ± 3 °C) was associated to the loss of the low molecular weight protein fraction and glycerol [45]. Finally, the maximum mass loss at temperatures above 300 °C (T_max_ = 322 ± 4 °C) was related to the decomposition of the larger size or tightly interacting protein fractions [10].

Regarding FG/PSP films, a general shift to higher initial degradation temperatures (T_ini_) was observed with increasing PSP content, showing FG/PSP30 a significant (*p* < 0.05) increase of about 40 °C compared to the FG control (Table 4), evidencing an enhancement on the thermal stability of the resulting films upon the addition of PSP at 30 wt. %. Moreover, a similar trend was observed for the oxidation induction time (OIT) values of the reinforced films, showing a significant increase (*p* < 0.05) with increasing PSP content. Regarding the oxidation onset temperature (OOT) no significant (*p* > 0.05) differences were observed between FG and formulations with PSP regardless of their concentration. This result could be attributed to the relatively high OOT values obtained with all these formulations, much higher than T_ini_ in all cases. Therefore, no significant (*p* > 0.05) differences should be expected for OOT, but the significant (*p* < 0.05) increase in OIT for formulations with PSP (32 ± 1 °C and 39 ± 3 °C for FG/PSP 10 and FG/PSP30, respectively) when compared to the OIT for FG films (14 ± 3 °C) is a clear indication of the antioxidant performance of PSP to protect FG against oxidation. As the overall result of the thermal analysis study, the addition of PSP at high contents seems to delay the thermal degradation process of the FG matrix. This behaviour was attributed to the effective cross-linking of gelatin with PSP, yielding a strong film network due to: a) the presence of phenolic compounds with high antioxidant activity, in agreement with the TPC, DPPH, FRAP and ABTS results; b) the presence of xylan and cellulose as main polysaccharides in PSP resulting in a highly crystalline structure that could act as heat barrier by delaying the heat flow arising from an exterior source [55]. These suggestions could be supported by previous studies in which the thermal stability of FG films was improved by the addition of ethanolic extracts from coconut husk [14], nano-SiO_2_ particles [12], cinnamon, clove and star anise [53]. Similar results were also reported for soy protein isolates and gelatin films reinforced with microcrystalline cellulose [56], where the thermal stability of films was improved by the addition of cellulose.

Differential scanning calorimetry (DSC) thermal parameters obtained for FG/PSP films are shown in Table 4. No significant differences (*p* > 0.05) in the glass transition temperature (T_g_) values were observed between FG and FG/PSP10 films whereas a significant (*p* < 0.05) lower value was obtained for FG/PSP30 film. This fact could suggest that the addition of PSP at 10 wt. % into the FG matrix can maintain the integrity of the obtained bio-composite but a 30 wt. % loading can promote the weakening of the interactions between polysaccharides and proteins due to a fibre excess [10]. The T_g_ value obtained for the FG film was in accordance with that previously reported for films obtained from salmon skin gelatin [44]. Figure 3B shows the calorimetric curves obtained for FG/PSP films showing three endothermic peaks. The fifirst peak around 100 °C was related to the loss of water and volatile compounds, in accordance with thermogravimetric analysis (TGA) results previously discussed. The second peak around 250 °C could be assigned to the devitrifification of blocks rich in α-amino acids; while the third one at 312 °C could be attributed to the devitrifification of imino acids blocks, such as hydroxyproline and proline, as already stated by other authors [15]. The addition of PSP results in the final disappearance of the third endothermic peak in FG/PSP10 and FG/PSP30 films (Table 4). These results are in agreement with those obtained by TGA and FTIR suggesting that PSP could possibly act as a heat barrier by reinforcing the film network. As a consequence, endothermic peaks related to the decomposition of the gelatin matrix tend to disappear.

### 2.5. Mechanical Properties of FG/PSP Films

Regarding mechanical properties (Table 5), no significant differences (*p* > 0.05) were observed for elongation at break (EAB) between the control film and FG/PSP10, while a drastic significant (*p* < 0.05) decrease was observed for the FG/PSP30 film, suggesting a reinforcing effect of PSP due to rigid PSP particles and good compatibility between the filler and the polymer matrix [29], which results in higher rigidity and stiffness at high PSP loading [54]. In addition, low interactions between PSP and the polymer matrix in FG/PSP30 could be responsible of the stress concentration and beginning of fracture resulting in a decrease in the EAB value. A similar behaviour was found by Ali et al. in starch films incorporating pomegranate peel with increasing EAB in composite films due to homogeneous dispersion and good interface of the filler [29]. However, no significant differences in EAB were observed by Hanani et al. in gelatin films added with pomegranate peel due to the development of structural discontinuities by the addition of pomegranate peel [28]. On the other hand, an increase in EAB was observed for κ-carrageenan films containing pomegranate seed oil due to the plasticizing effect of the additive increasing the chain mobility of the composite films [30].

Although elastic modulus values were not significantly modified (*p* > 0.05) by the addition of PSP, an insignificant (*p* > 0.05) increase in tensile strength (TS) was observed in FG/PSP films with increasing PSP concentration. Some authors have related a similar TS improvement in gelatin films with the addition of polysaccharides present in pomegranate residues, such as pectin, and the interaction of polymer side chains with hydroxyl groups of phenolic compounds. Thus, OH groups can contribute to the formation of intermolecular hydrogen bonds between phenolic compounds and gelatin, resulting in higher TS [28]. These results are in accordance with the carbohydrate composition of PSP, where abundant hydroxyl groups and oxygen atoms from the xylan and cellulose polysaccharides could interact with the FG matrix, as it was previously discussed in the FTIR section.

### 2.6. Solubility and Barrier Properties of FG/PSP Films

A significant (*p* < 0.05) decrease in films solubility to water was observed with increasing concentration of PSP from 83 ± 3% for the FG film to 16 ± 4% for FG/PSP30 (Table 5), which may be attributed to an increase in electrical compatibility between the gelatin protein and polysaccharides arising from the promoted intermolecular interactions, leading to more effective moisture barrier properties [30]. A similar behaviour was observed by Hanani et al. for gelatin films functionalized with pomegranate peel at 2–5 wt. % and this trend was linked to the cross-linking effect of phenolic compounds and sugars present in pomegranate peel and to the presence of insolubilized parts of the pomegranate peel powder remaining at the end of the solubility test [28]. The higher resistance to water observed for FG/PSP films could be also ascribed to the more hydrophobic components present in PSP, mainly lignin, as it was described in the carbohydrate analysis, ensuring low water affinity throughout the film structure and thus increasing water resistance. According to some authors, the non-polar components of hydrophobic agents could reduce the availability of hydroxyl groups, which interact with water, resulting in decreased moisture absorption of hydrocolloids-based composite films [45].

Finally, the addition of PSP did not significantly affect (*p* > 0.05) the gas barrier properties of composite films, such as oxygen transmission rate (OTR) and water vapour permeability (WVP) (Table 5). Nevertheless, OTR values showed a slight increasing trend with the addition of PSP, suggesting the formation of alternative pathways or cracks into the polymer matrix due to the presence of PSP as it was reported in a previous work for almond skin fibres [48]. A different behaviour in WVP was observed by Hanani et al. when incorporating pomegranate peel to gelatin films showing a significant increase in WVP values which was attributed to an incomplete dissolution of pomegranate peel in gelatin films which eliminated the strong bonds influencing the passage of water vapour through the film [28]. So, in this case, it can be assumed that a uniform distribution of PSP into gelatin was obtained for FG/PSP films according to the barrier properties obtained, in accordance with SEM results.

### 2.7. Antimicrobial Activity of FG/PSP Films

**Figure 4:**
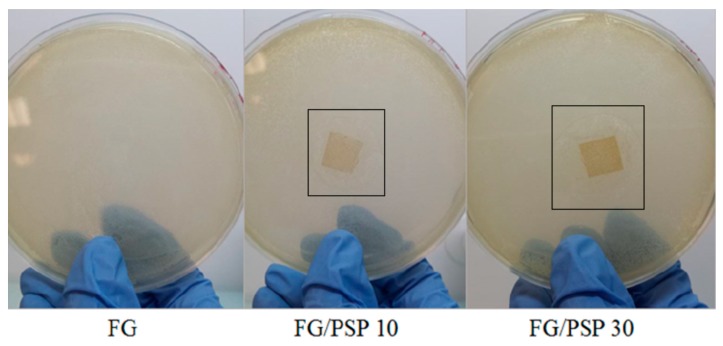
Pictures of the antimicrobial tests performed for all the studied formulations against *S. aureus* strains.

The antimicrobial activity results of FG/PSP films against *S. aureus* and *Salmonella enterica* is presented in Table 5. No inhibition zone was observed for the FG control against the bacteria tested. FG films including PSP showed a clear antimicrobial performance against *S. aureus* with significant (*p* < 0.05) increase in the observed inhibition zone with increasing PSP concentration from 10 to 30 wt. % (Figure 4). These results are clearly indicative of the high antimicrobial performance of pomegranate seed and peel extracts, as reported by several authors [57,58]. This behaviour can be related to the polyphenolic compounds present in PSP in accordance with previous results linking the antibacterial activity of pomegranate fruit and peel extracts with their total polyphenols content [59,60,61], being phenols, tannins and flavonoids the major active compounds responsible for this activity. However, it is difficult to attribute the antibacterial activity to a single or particular constituent when a complex mixture of bioactive compounds is present, such as in PSP. Therefore, antibacterial effects may be attributed to the combined action of various bioactive compounds which can provoke the bacterial death following diverse action mechanisms. Several of them could act on specific targets simultaneously, and they have been reported to explain the antimicrobial action of main polyphenols present in pomegranate peel, which could be associated with: a) precipitation of cell membrane proteins incurring in cell lysis; b) the inhibition of microbial enzymes through reaction with sulfhydryl groups; or c) non-specific interactions with proteins. Likewise, phenolic compounds may react with protein sulfhydryl groups making them unavailable for microbial growth thereby generating phenolic toxicity. The antibacterial activity of phenolic acids and flavonoids may be attributable to the cytoplasmic membrane damage caused by perforation and/or a reduction in membrane fluidity [24]. Finally, some researchers have suggested that the primary mechanism of action of plant-based antimicrobials with varying compositions could be related to the hydrophobic nature of their various components, which allows them to partition in the lipids of cell membranes, ultimately rendering them more permeable [62]. In summary, the mechanism of action may depend on the chemical composition of the active compounds and their antimicrobial activity could not be attributable to a unique mechanism but is instead a cascade of reactions involving the entire bacterial cell.

However, no inhibition zone was observed for FG/PSP films against *Salmonella enterica* (Table 5). This fact could be related to the Gram-negative character of this bacterial strain, which possess an additional external lipopolysaccharide membrane, contributing to being more resistant to natural extracts with antimicrobial activity [63]. In this sense, it has been reported than small hydrophilic solutes are able to pass through this outer membrane via abundant porin proteins that serve as hydrophilic transmembrane channels, and this is one of the reasons why Gram-negative bacteria are relatively resistant to hydrophobic antibiotics and toxic drugs [64]. This fact could be related to the previously discussed high resistance to water observed for FG/PSP films which was ascribed to the hydrophobic components present in PSP, mainly lignin, as it was described in the carbohydrate analysis. So, the non-polar components of hydrophobic agents present in PSP could reduce the availability of hydroxyl groups to penetrate the membrane cell of *Salmonella enterica*.

The obtained results are in agreement with previous works reporting higher antimicrobial activity of pomegranate against *S. aureus* [65]. Antimicrobial activity of biodegradable films incorporating pomegranate peels and seed oil has been found to be more effective against Gram-positive bacteria (*S. aureus*, *L. monocytes*) than against Gram-negative bacteria (*E. Coli*, *Salmonella enterica*) [28,29,30] and this fact was linked to the more effective action of phenolic compounds against Gram-positive microorganisms. Yuan et al. also found a higher resistance of Gram negative bacteria *E. coli* against chitosan films containing carvacrol and pomegranate peel extract, as the cell walls of Gram-negative bacteria may prevent that active components can reach the cytoplasmic membrane [61]. The antimicrobial activity of pomegranate peel has been reported to be stronger than the red and white seeds, juice and whole fruit [65]. In addition, the effectiveness of the antimicrobial activity of film samples containing pomegranate has been strongly related to the retention and diffusivity mechanism of active compounds in the polymer matrix [30].

## 3. Materials and Methods

### 3.1. Chemicals and Reagents

High molecular weight gelatin from cold deep-water fish (G2963A) was obtained from Healan Ingredients (York, UK). Glycerol, sodium carbonate, glacial acetic acid, ferric chloride, potassium persulfate, ethanol (HPLC grade), *n*-hexane (GC grade) were obtained from Panreac (Barcelona, Spain). Gallic acid, Trolox (6-hydroxy-2,5,7,8-tetramethylchroman-2-carboxylic acid), Folin-Ciocalteu reagent, DPPH, 2,4,6-tripyridyl-*S* triazine (TPTZ) and ABTS were acquired from Sigma-Aldrich (St. Louis, MO, USA). All solvents and chemicals used were of analytical grade. For antimicrobial tests, *Staphylococcus aureus* subsp. *aureus* (CECT 59) and *Salmonella enterica* subsp. *enterica* (CECT 443) were acquired from the Culture Type Spanish Collection (CECT, Valencia, Spain). Mueller-Hinton agar was purchased from Bio-Rad Laboratories, Inc (Hercules, CA, USA).

### 3.2. Pomegranate seed juice industrial by-product (PSP) preparation

**Figure 5:**
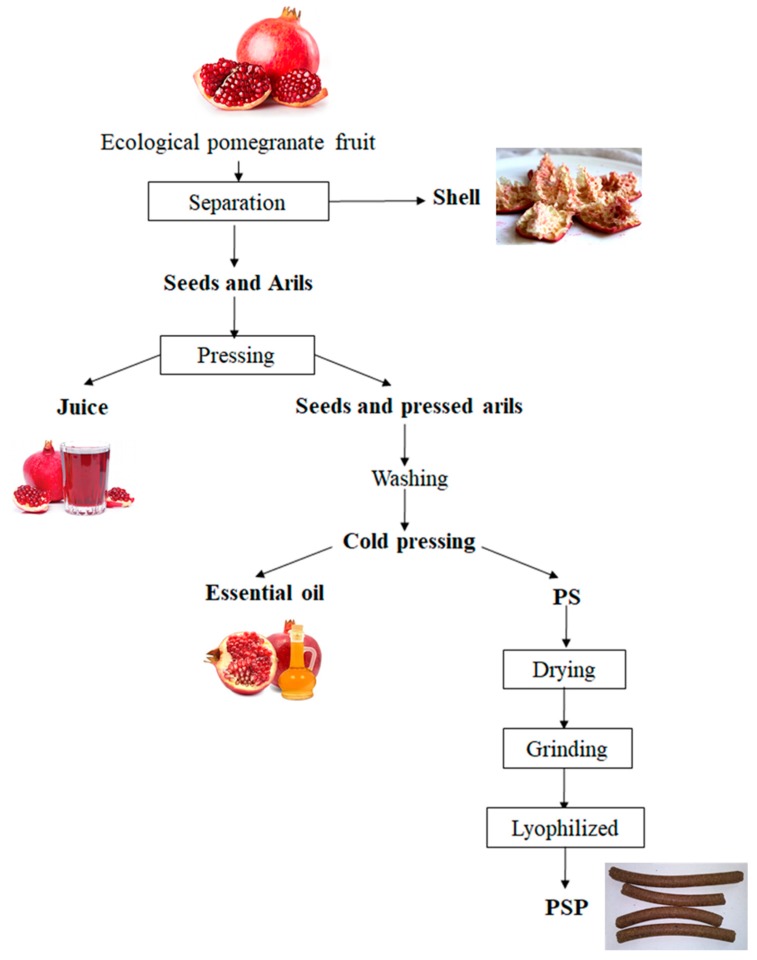
General scheme of PSP production from pomegranate industrial juice manufacturing.

Pressed pomegranate (*Punica granatum* L.) seeds and arils (PS) were collected as the final by-product obtained after pomegranate industrial juice manufacturing from a Spanish company, as indicated in Figure 5. PS was dried in an oven (50 °C, 24 h) and grinded into a fine powder with a high-speed rotor mill (Ultra Centrifugal Mill ZM 200, RETSCH, Haan, Germany) equipped with a 80 µm sieve. The PS powder fraction (PSP) obtained was lyophilized (Alpha 1–3 LDplus, Martin Christ Gefriertrocknungsanlagen GmbH, Osterode, Germany) and stored at −20 °C protected from light for further use.

### 3.3. PSP Characterization

#### 3.3.1. Chemical Composition Analysis

Total ash, moisture and fat contents of PSP were determined by following the methods proposed by the Association of Official Analytical Chemists (AOAC) guidelines [66]. All analyses were performed in triplicate and mean results were expressed on a dry-weight basis (g/100 g DW). For moisture and ash content determination, 1.000 ± 0.001 g of PSP was introduced in an oven (Selecta, Barcelona, Spain) at 105 °C and 550 °C, respectively, until constant weight. Fat content was calculated by weight loss after 4 h extraction of 2.000 ± 0.001 g of PSP with *n*-hexane under reflux in a Soxhlet apparatus.

#### 3.3.2. Carbohydrate Composition and Glycosidic Linkage Analysis

The carbohydrates composition of PSP was determined, in triplicate, after trifluoroacetic acid (TFA) and sulfuric acid hydrolysis. For TFA hydrolysis, 1 mg of freeze-dried sample was incubated with 1 mL of 2 M TFA for 3 h at 120 °C. Samples were then dried under air stream and dissolved with deionized water. Sulfuric acid hydrolysis was performed by adding 250 μL of 72% H_2_SO_4_ to 4 mg of sample and further incubation at room temperature for 3 h. Then, deionized water was added to dilute the solution to approx. 1.2–1.3 M sulfuric acid in a tube with further incubation at 100 °C for 3 h. Monosaccharides were analysed using high performance anion exchange chromatography with pulsed amperometric detection (HPAEC-PAD) with an ICS-3000 system (Dionex, ThermoFisher Scientific, Waltham, MA, USA) equipped with a CarboPac PA1 column (250 mm length and 4 mm diameter, Dionex). Inositol was added to all samples as an internal standard prior to hydrolysis.

For glycosidic linkage analysis, freeze-dried PSP samples (5 mg) were reduced with sodium borodeuteride (NaBD_4_) after activation with carbodiimide to label the uronic acids present in the fractions, following the protocol reported by Kim and Carpita [67]. Samples (1 mg, three replicates) were further swelled in anhydrous dimethyl sulfoxide (DMSO) for 16 h at 60 °C and methylated five times in the presence of NaOH/CH_3_I [68] to ensure complete methylation. Samples were then hydrolysed with 2 M TFA at 120 °C for 2 h, reduced with sodium borohydride (NaBH_4_) and acetylated with acetic anhydride in pyridine [69]. The obtained permethylated alditol acetates (PMAAs) were separated and analysed by gas chromatography (HP-6890, Agilent Technologies, Santa Clara, CA, USA) coupled to an electron-impact mass spectrometer (HP-5973, Agilent Technologies) with a SP-2380 capillary column (30 m × 0.25 mm i.d., Sigma–Aldrich). The temperature program was set from 160 to 210 °C at 1 °C/min heating rate. The mass spectra of fragments obtained from PMAAs were identified by comparing them with those of reference polysaccharide derivatives [70]. Quantification was based on the effective carbon response of each compound and it was further corrected with the total monosaccharide composition obtained from the carbohydrate composition analysis.

#### 3.3.3. Total Phenolic Content

TPC was determined, in triplicate, by following the Folin-Ciocalteu colorimetric method [71]. For this purpose, 65 g of grinded PSP were extracted with 260 mL ethanol (minimum solvent volume allowing the complete mixture of the sample [40]). Samples were stirred for 24 h at ambient temperature in the dark. The extraction mixture was then filtered and evaporated at 40 °C in the dark obtaining a brown sticky residue with 2.6 ± 0.3% extraction yield. This residue was immediately dissolved with ethanol (34 mg/mL). Deionized water (790 µL) was added to 10 µL of the PSP extract. Then, 50 µL of Folin–Ciocalteu reagent were incorporated to the mixture, which was stirred with a Vortex and incubated for 3 min. 150 µL of 20% aqueous Na_2_CO_3_ solution were then added and the absorbance was measured at 760 nm after 180 min of incubation in the dark using deionized water as a blank and a Biomate-3 UV/VIS spectrophotometer (Thermospectronic, Mobile, AL, USA). Gallic acid was used as the reference standard and TPC was expressed as mg gallic acid equivalent (GAE) per gram of PSP sample (mg GAE/g).

#### 3.3.4. Antioxidant Activity

Three methods based on electron transfer reactions were used in this study to evaluate the antioxidant activity of PSP. All tests were done in triplicate.

##### DPPH Radical Scavenging Assay

An aliquot of 10 µL of diluted PSP extracts (0–12 mg/mL) was added to 1 mL of 30 mM ethanolic solution of the free radical DPPH. The absorbance was monitored spectrophotometrically at 517 nm after 15 min incubation at ambient temperature. The radical DPPH scavenging capacity (%) and the extract concentration required to cause 50% reduction in the initial DPPH concentration (IC_50,_ mg/mL) were determined as reported elsewhere [40].

##### ABTS Free Radical Scavenging Assay

An aliquot of 10 µL of diluted PSP extracts (0–34 mg/mL) was added to 1 mL of the ABTS^+^ working solution. The absorbance inhibition at 734 nm was determined after 6 min at ambient temperature as a function of the antioxidant concentration. The extract concentration required to cause 50% reduction in the initial ABTS concentration (IC_50,_ mg/mL) was also determined.

##### Ferric Reducing Antioxidant Power (FRAP).

The capacity of PSP extracts to reduce ferric ions was assessed by the ferric reducing ability of plasma (FRAP) method [71]. Measurements were performed at 593 nm after 30 min incubation in the darkness at 37 °C. FRAP results were expressed as mM of Trolox equivalent per gram of sample (mM TE/g).

### 3.4. Preparation of Fish Gelatin-Based Films Incorporating PSP (FG/PSP)

Gelatin films were prepared by solvent-casting at room temperature. PSP was previously hydrated for 60 min in distilled water under magnetic stirring (100 rpm). Fish gelatin (8%, *w/w*) was dissolved in the PSP/water solution under stirring for 20 min. Glycerol (25%, *w/w* based on fish gelatin) was further added to the mixture and stirred for 10 min. Three different formulations were obtained by adding 0, 10 and 30 wt. % PSP (based on the gelatin weight) into the film solution. Film-forming solutions were sonicated for 30 min to remove air bubbles and they were finally cast onto Petri dishes at 50% relative humidity (RH) and 23 ± 1 °C in a climate chamber (Dycometal, Barcelona, Spain) for 24 h. The obtained films were coded as FG (without any added PSP as the control), FG/PSP 10 and FG/PSP 30; where the number corresponds to the percentage of PSP added by weight, respectively.

### 3.5. Films Characterization

#### 3.5.1. Film Thickness

The average thickness of the obtained films was measured by using a 293 MDC-Lite Digimatic Micrometer (Mitutoyo, Kanagawa Prefecture, Japan) at five random positions, after 48 h of film conditioning at 50% RH and 23 °C.

#### 3.5.2. Light Transmission and Transparency

Ultraviolet (UV) and visible optical density (OD) values of fifilms were measured, in triplicate, at 280 and 600 nm, respectively, using a Biomate-3 UV-Vis spectrophotometer, according to the method described by Hosseini et al. [15], using the following equations:

UV OD = A_280_/X  Vis OD = A_600_/X (1)

where A_280_ and A_600_ are the absorbance at 280 and 600 nm, respectively, and X is the film thickness (mm). The greater transparency value represents the lower OD.

#### 3.5.3. Scanning Electron Microscopy (SEM)

The surface morphology of PSP particles and FG/PSP films was studied using a JSM-840 scanning electron microscope (JEOL, Peabody, MA, USA) under an accelerating voltage of 15 kV. Magnifications of 1500× and 1000× were used for PSP and FG/PSP films, respectively. Before analysis, samples were coated with gold under vacuum using a SCD 004 Balzers sputter coater (Bal Tec. AG, Furstentum, Lichtenstein) to increase their electrical conductivity.

#### 3.5.4. Attenuated Total Reflectance-Fourier Transform Infrared (ATR-FTIR) Spectroscopy

An IFS 66 FTIR spectrometer (Bruker Analitik, Ettlingen, Germany) equipped with a DTGS KBr detector and Golden Gate single reflection diamond ATR accessory (incident angle 45°) was used. PSP (2.00 ± 0.01 mg) and FG/PSP films (1 × 1 mm^2^) were directly placed on the ATR crystal area for measurements. Spectra were recorded in the absorbance mode from 600–4000 cm^−1^, using 64 scans and 4 cm^−1^ resolution, and corrected against the background spectrum of air. Prior to analysis, FG/PSP fifilms were conditioned in a climate chamber at 50% RH and 23 ± 1 °C for 48 h whereas PSP was dehydrated at 40 °C for 48 h in an oven. Three replicates were obtained for each sample.

#### 3.5.5. Thermogravimetric Analysis (TGA)

A thermogravimetric microbalance TGA/SDTA 851 Mettler Toledo (Schwarzenbach, Switzerland) was used to evaluate the thermal stability of PSP and FG/PSP films. Samples (6.0 ± 0.1 mg) were heated from 30 to 700 °C at 10 °C/min under N_2_ atmosphere (50 mL/min). Analyses were performed in triplicate. The initial degradation temperature, T_ini_ (°C), and temperature of maximum degradation, T_max_ (°C), were determined according to Valdés et al. [48].

#### 3.5.6. Differential Scanning Calorimetry Analysis (DSC)

Differential scanning calorimetry (DSC) analysis of FG/PSP films was carried out, in triplicate, with a TA DSC Q-2000 instrument (New Castle, DE, USA). Film samples were initially conditioned in a climate chamber at 50% RH and 23 ± 1 °C for 48 h. 5.00 ± 0.01 mg of samples were placed into aluminium pans, sealed and scanned over a range of 25–350 °C with a heating rate of 10 °C/min under N_2_ atmosphere (50 mL/min). Peak temperature values of samples were determined as the maximum of the observed endothermic transitions. The glass transition temperature (T_g_) was determined by heating from −60 °C to 90 °C (3 min hold), cooling to −60 °C (3 min hold) and heating to 90 °C (3 min hold), all steps at 10 °C/min. The T_g_ values, defined as the midpoint of the change in the heat capacity, were calculated from the second DSC heating scan [44].

The thermo-oxidative performance of FG/PSP films, by means of oxidation onset temperature (OOT) and oxidation induction time (OIT) parameters, was determined by weighing 4.00 ± 0.01 mg of each sample. For OOT analysis, samples were heated up to 200 °C under oxygen atmosphere at 10 °C/min, before the calculation of the degradation onset, which was identified by the initiation of an exothermic process in the DSC calorimetric curve. For OIT determination, samples were heated up to 195 °C under nitrogen atmosphere at 100 °C/min. At this temperature, oxygen was inserted into the DSC chamber and the degradation onset was identified by the initiation of an exothermic process in the DSC calorimetric curve.

#### 3.5.7. Mechanical Properties

Before testing, FG/PSP films were equilibrated for 48 h at 23 °C and 50% RH. Mechanical properties were determined at ambient temperature using a 3340 Series Single Column System Instron Instrument, LR30K model (Fareham Hants, UK) equipped with a 100 kN load cell. An initial grip separation of 50 mm and a crosshead speed of 20 mm/min were used. Three tensile parameters (elongation at break (%), tensile strength (MPa) and elastic modulus (MPa)) were obtained from the stress-strain curves following the ASTM D882-09 standard. Five replicates were performed for each formulation and average values were reported.

#### 3.5.8. Barrier Properties

FG/PSP films (2 × 2 cm^2^) were firstly conditioned in a climate chamber at 50% RH and 23 ± 1 °C for 48 h. The water solubility of films was determined as described by Hosseini et al. [15]. Water vapour permeability (WVP) was measured by using the desiccant method (CaCl_2_) and oxygen transmission rate (OTR) tests were performed with an oxygen permeation analyser (8500 model Systech Instruments, Metrotec S.A, Lezo, Spain) as reported elsewhere [48].

#### 3.5.9. Antimicrobial Activity

The antimicrobial activity of PSP/FG films was evaluated, in triplicate, by the agar diffusion method against two microorganisms: Gram positive *S. aureus* and Gram negative *S. enterica* bacterial strains, commonly associated with spoilage in refrigerated foods. Sterilized fifilms, under ultraviolet light for 10 min, were cut into 1 × 1 cm^2^ discs and placed onto Muller Hinton Agar plates, previously seeded with 0.1 mL of inoculum by swabbing with approximately 10^5^ CFU/mL of the tested bacteria, previously standardized using the McFarland scale [62]. The diameter of the inhibition zone (mm) around the disc was measured after 24 h of incubation at 37 °C.

### 3.6. Statistical Analysis

Statistical analysis of experimental data was performed by one-way analysis of variance (ANOVA) using SPSS 15.0 (Chicago, IL, USA) and expressed as mean ± standard deviation. Differences between average values were assessed based on the Tukey test at a confidence level of 95% (*p* < 0.05).

## 4. Conclusions

This study reported, for the first time, the development and characterization of fish gelatin edible films containing different loadings of PSP residue as reinforcing and antimicrobial agent. The carbohydrate linkage profile of PSP underlined the presence of two main polysaccharides, xylan and cellulose, contributing to reduce water solubility of the FG/PSP composite films. PSP samples showed high antioxidant performance as stated by their total phenolic content and antioxidant activity obtained results. The potential of this residue as an antimicrobial additive against Gram positive bacteria *S. aureus* in FG films was also highlighted. The best overall performance considering all the studied properties was found for films with 10 wt. % PSP loading. The addition of this residue enhanced the films stiffness, particularly for FG/PSP30 making this formulation too stiff for films processing, and provided good ultraviolet and visible light barrier properties at both PSP loadings, resulting in improvement in opacity, which could be useful for food with high lipid content. The addition of PSP also improved the water resistance of the developed bio-composites due to the hydrophobic character of the residue at both concentrations. In conclusion, the FG/PSP 10 wt. % formulation could be an interesting ecological-friendly material to be used in films for active food packaging to prevent the oxidative deterioration of refrigerated or chilled foodstuff in which transparency is not an issue. This strategy can also increase the added-value potential of pomegranate agricultural wastes obtained from pressed seeds and arils in the food industry. Finally, the addition of PSP to fish gelatin could be considered a sustainable method to reduce huge amounts of pomegranate and fish wastes, contributing to the well-known circular economy concept; while prolonging at the same time the shelf-life of food packaged products.

## Figures and Tables

**Table 1:** Carbohydrate composition of PSP, calculated by TFA and sulfuric hydrolysis (n = 3, mean ± SD).

Sugar Unit	Relative Abundance (wt. %)	
	TFA Hydrolysis	Sulfuric Hydrolysis
Fucose	Traces	traces
Arabinose	6.74 ± 0.09	3.70 ± 0.04
Rhamnose	2.25 ± 0.11	0.71 ± 0.10
Galactose	4.52 ± 0.35	2.73 ± 0.05
Glucose	10.2 ± 1.94	48.45 ± 0.41
Xylose	66.74 ± 1.47	36.92 ± 0.49
Mannose	3.23 ± 0.34	2.05 ± 0.42
Galacturonic acid	5.41 ± 1.30	4.25 ± 0.10
4-*O*-Me-Glucuronic acid	1.65 ± 0.83	1.18 ± 0.26
**Carbohydrate Type**	**Relative Abundance (wt. %)**	
Cellulose	38.3 ± 2.4	
Xylan	43.2 ± 3.1	
Pectins	11.4 ± 3.4	

**Table 2:** Carbohydrate linkage analysis of PSP (*n* = 3, mean ± SD).

Linkage	Structural Units Deduced	Relative Abundance (% mol)
t-Ara*f*	Ara*f*-(1 →	3.16 ± 0.99
3-Ara*p*	→ 3) Ara*p*-(1 →	0.25 ± 0.07
5-Ara*f*	→ 5) Ara*f*-(1 →	2.27 ± 0.84
3,5-Ara*f*	→ 3,5) Ara*f*-(1 →	1.06 ± 0.28
Total Ara		6.74 ± 2.27
t-Xyl*p*	Xyl*p*-(1 →	2.53 ± 1.16
4-Xyl*p*	→ 4)-Xyl*p*-(1 →	62.6 ± 3.0
2,4-Xyl*p*	→ 2,4)-Xyl*p*-(1 →	1.29 ± 0.38
3,4-Xyl*p*	→ 3,4)-Xyl*p*-(1 →	0.31 ± 0.06
Total Xyl		66.7 ± 4.6
t-Glc*p*	Glc*p*(1 →	0.23 ± 0.13
4-Glc*p*	→ 4) Glc*p*-(1 →	9.25 ± 0.61
4,6-Glc*p*	→ 4,6)-Glc*p*-(1 →	0.28 ± 0.17
3,4-Glc*p*	→ 3,4)-Glc*p*-(1 →	0.44 ± 0.13
Total Glc		10.2 ± 1.1
t-Man*p*	Man*p*(1 →	0.21 ± 0.10
4-Man*p*	→ 4) Man*p*-(1 →	2.53 ± 0.55
4,6-Man*p*	→ 4,6) Man*p*-(1 →	0.49 ± 0.17
Total Man		3.23 ± 1.00
t-Gal*p*	Gal*p*-(1 →	1.67 ± 0.55
2-Gal*p*	→ 2) Gal*p*-(1 →	0.73 ± 0.01
4-Gal*p*	→ 4) Gal*p*-(1 →	0.17 ± 0.08
6-Gal*p*	→ 6) Gal*p*-(1 →	0.58 ± 0.09
2,4-Gal*p*	→ 2,4) Gal*p*-(1 →	0.53 ± 0.57
3,6-Gal*p*	→ 3,6) Gal*p*-(1 →	0.84 ± 0.35
Total Gal		4.52 ± 1.64
t-Rha*f*	Rha*f*-(1 →	1.14 ± 0.35
2-Rha*f*	→ 2) Rha*f*-(1 →	0.88 ± 0.20
2,4-Rha*f*	→ 2,4) Rha*f*-(1 →	0.23 ± 0.03
Total Rha		2.25 ± 0.68
t-Gal*p*A	Gal*p*A-(1 →	1.94 ± 0.22
4-Gal*p*A	→ 4) Gal*p*A-(1 →	2.71 ± 0.32
3,4-Gal*p*A	→ 3,4) Gal*p*A-(1 →	0.76 ± 0.21
Total GalA		5.41 ± 0.77
t-MeGlc*p*A	MeGlc*p*A(1 →	1.65 ± 0.07
Total MeGlcA		1.65 ± 0.07

**Table 3:** Optical density (*n* = 3) values of fish gelatin films with PSP (mean ± SD).

		Film Samples		
		FG	FG/PSP10	FG/PSP30
Optical density (cm^−1^)	λ = 280 nm	7.8 ± 2.3 ^a^	18.9 ± 2.2 ^b^	24.6 ± 0.9 ^c^
	λ = 600 nm	0.45 ± 0.03 ^a^	1.2 ± 0.4 ^b^	1.8 ± 0.2 ^c^

Different superscripts (a, b, c) within the same line indicate statistically significant different values (*p* < 0.05).

**Table 4:** Thermal parameters obtained for bio-composite films (*n* = 3, mean ± SD).

Thermal Property	FG	FG/PSP10	FG/PSP30
T_ini_ (°C)	125 ± 8 ^a^	133 ± 6 ^a^	166 ± 6 ^b^
T_max1_ (°C)	259 ± 3 ^a^	260 ± 4 ^a^	256 ± 4 ^a^
T_max2_ (°C)	322 ± 4 ^a^	325 ± 4 ^a^	327 ± 1 ^a^
OOT (°C)	236 ± 1 ^a^	237 ± 2 ^a^	238 ± 2 ^a^
OIT (min)	14 ± 3 ^a^	32 ± 1 ^b^	39 ± 3 ^c^
T_g_ (°C)	42 ± 1 ^a^	39 ± 1 ^a^	30 ± 1 ^b^
T_p1_ (°C)	250 ± 4 ^a^	254 ± 1 ^a^	247 ± 1 ^a^
T_p2_ (°C)	312 ± 1 ^a^	nd	nd

nd: not detected. Different superscripts (a, b, c) within the same line indicate statistically significant different values (*p* < 0.05).

**Table 5:** Mechanical (*n* = 5), barrier (*n* = 3) and antimicrobial (*n* = 3) properties of fish gelatin films with PSP (mean ± SD).

Property		Film Samples		
		FG	FG/PSP10	FG/PSP30
Mechanical	Elongation at break (%)	216 ± 64 ^a^	191 ± 52 ^a^	2.0 ± 0.6 ^b^
	Elastic modulus (MPa)	10 ± 3 ^ab^	8 ± 1 ^a^	12 ± 2 ^b^
	Tensile strength (MPa)	149 ± 45 ^a^	161 ± 23 ^a^	242 ± 90 ^a^
Barrier	OTR.e (cm^3^ mm m^−2^ day)	454 ± 75 ^a^	568 ± 80 ^a^	577 ± 71 ^a^
	WVP × 10^−13^ (kg m Pa^−1^ s^−1^ m^−2^)	1.55 ± 0.04 ^a^	1.63 ± 0.02 ^a^	1.53 ± 0.02 ^a^
	Solubility (%)	83 ± 3 ^a^	44 ± 2 ^b^	16 ± 4 ^c^
Antimicrobial (inhibition zone)	*S. aureus* (cm)	nd	2.6 ± 0.2 ^a^	3.3 ± 0.1 ^b^
	*S. enterica* (cm)	nd	nd	nd

nd: not detected. Different superscripts (a, b, c, ab) within the same line indicate statistically significant different values (*p* < 0.05).
